# Effects of different anesthesia methods on postoperative immune function in patients undergoing gastrointestinal tumor resection

**DOI:** 10.1038/s41598-023-27499-2

**Published:** 2023-01-05

**Authors:** Yan Zhang, JunJun Lu, MingZhe Qin, MengDa Xu, WenJun Luo, BiXi Li, XiaoYang Song, Xiang Zhou

**Affiliations:** Department of Anesthesiology, General Hospital of Central Theater Command of PLA, Wuhan, 430070 China

**Keywords:** Cancer, Immunology, Medical research

## Abstract

To investigate the effects of different anesthetic methods on postoperative immune function in patients undergoing gastrointestinal tumor resection. Ninety patients undergoing laparoscopic gastrointestinal tumor resection were divided into 3 groups. Patients in the GA group were anesthetized by total intravenous anesthesia. The GE group was anesthetized by general anesthesia combined with epidural anesthesia. The GN group was anesthetized by general anesthesia combined with bilateral Transversus Abdominis Plane block (TAP) and rectus sheath nerve blocks. General anesthesia is total intravenous anesthesia in all three groups. Blood samples were taken to test the changes of peripheral lymphocyte subtype analysis, and levels of plasma cortisol, epinephrine, norepinephrine. Also, the dosage of anesthetic drugs, recovery time, and visual analog scale (VAS) scores were recorded. Postoperative immune indexes, including CD4 count, CD8 count, B, and NK cells, in the GE group were significantly higher than those in NA and GA groups (*P* < 0.01). Perioperative stress indices, including epinephrine levels, norepinephrine level and aldosterone level, in the GE group were significantly lower than in the GA group and GN group (*P* < 0.01). The intraoperative/total sufentanil dosage and remifentanil dosage in the GE group were significantly lower than those in the GA and GN groups (*P* < 0.01). The VAS scores in the GE group were significantly better than those in GA and GN groups (*P* < 0.01). General anesthesia combined with epidural anesthesia attenuates the increase in inflammatory mediators. Its possible mechanisms include reducing perioperative stress response and reducing perioperative opioid use.

## Introduction

Surgical stress response^1,2^ and general anesthetics^3–5^ can adversely affect the postoperative immune function of patients by reducing the number of immune cells, including NK and CD4 cells, and increasing the risk of tumor cell proliferation and metastasis, which will lead to poor prognosis in patients undergoing tumor resection^3,5^. In modern society, the anesthesiologist’s role is gradually expanding. The discipline is no longer solely tasked with providing sedation, analgesia and muscle relaxation intraoperatively, but has expanded into the perioperative period. Maintaining the immune function of patients during the perioperative period is an emerging area for the contemporary anesthesiologist.

General anesthesia (GA) combined with epidural anesthesia is a common method of anesthesia, commonly used in thoracic and abdominal major operations. Reports have indicated that general anesthesia combined with epidural anesthesia can inhibit the level of surgical stress, reduce the release of catecholamine hormone, and improve the prognosis of patients^6,7^. Recently, ultrasound-guided nerve block technology has gradually become popular^8,9^, which has a trend to replace the traditional method of general anesthesia combined with epidural anesthesia. However, the different effects of total intravenous anesthesia (TIVA), GA combined with epidural anesthesia, or peripheral nerve block on postoperative immune function are still unknown.

To further investigate the effects of different anesthetic methods on postoperative immune function, we designed this clinical trial to observe the effects of GA, GA combined with nerve block, or GA combined with epidural anesthesia on immune cell function, stress response, perioperative drug dosage, the analgesic effects and recovery quality. We hypothesis that GA combined with epidural anesthesia can better protect postoperative immune function. The primary aim of the study were peripheral lymphocyte subtype analysis, including CD4, CD8, B lymphocyte and NK cell count. Secondary aim was the analysis of levels of plasma cortisol, epinephrine, and norepinephrine.

## Methods

### Patients and grouping

This single-center, prospective clinical study was approved by the Ethics Committee of the General Hospital of Central Theater Command of People’s Liberation Army (2021-005-7) and passed the clinical trial registration application (ChiCTR2100047982, 28/06/2021) at http://www.chictr.org.cn/index.aspx. All methods were performed in accordance with the relevant guidelines and regulations. Informed consent was obtained from all participants or their legal guardians. The research was performed in accordance with the Declaration of Helsinki.

Ninety patients undergoing elective laparoscopic gastrointestinal tumor resection from July 1, 2021, to January 31, 2022, gave consent to participate. All patients were divided into 3 groups using a computerized random number generator (Random Number Generator Software v2.1.0, HeFei, China): the total intravenous anesthesia/general anesthesia group (GA group, n = 30), general anesthesia combined with the epidural anesthesia group (GE group, n = 30) and general anesthesia combined with peripheral nerve block group (GN group, n = 30).

### Inclusion and exclusion criteria

Inclusion criteria: (1) elective laparoscopic gastrointestinal tumor resection; (2) ASA(American Society of Anesthesiologists) class I–III; (3) age 18–70 years old. Exclusion criteria: (1) Refusal to participate in the clinical trial; (2) immune system diseases (HIV, systemic lupus erythematosus); (3) severe liver and kidney diseases; (4) drug and alcohol abuse history; (5) allergy to drugs included in the study protocol; (6) coagulation dysfunction; (7) skin infections at the site of neuraxial and peripheral nerve block cannulation; (8) other contraindications of epidural and nerve block anesthesia.

### Anesthesia method

Patients in the GA group were anesthetized by total intravenous anesthesia, and the induction drugs included midazolam 0.1 mg/kg, etomidate 2–3 mg/kg, sufentanil 0.5–1 µg/kg, and cisatracurium besilate 2 mg/kg. Propofol and remifentanil were continuously injected to maintain the BIS value (186-1100, COVIDIEN, USA) at 40–60, and cisatracurium besilate was given intermittently to maintain muscle relaxation. The patient controlled intravenous analgesia pump (Sufentanil 0.25 µg/kg with Tropisetron 4 mg) was used to alleviate postoperative pain. Patients in the GE group were anesthetized by general anesthesia combined with epidural anesthesia. Epidural puncture and catheterization were performed before general anesthesia under ultrasound guidance: T9–T10 for gastric surgery, T11–T12 for colon surgery, and T12–L1 for rectal surgery. Five milliliters of 1% lidocaine were given as the experimental dose, and 10 ml of 0.2% ropivacaine was injected from the epidural catheter; the anesthetic effect was measured 15 min later. Then, 6 ml of 0.2% ropivacaine was added every hour through the epidural catheter, and 0.15% ropivacaine with Sufentanil 0.15 µg/kg was given as patient-controlled epidural analgesia after the operation. Before general anesthesia, patients in the GN group underwent bilateral Transversus Abdominis Plane blocks (TAP) and rectus sheath nerve blocks with 0.375% ropivacaine (10 ml for each point for a total of 40 ml) under ultrasound guidance; the block range was measured 15 min later. After the operation, patients were given patient-controlled analgesia with an intravenous analgesia pump (Sufentanil 0.25 μg/kg with Tropisetron 4 mg). General anesthesia induction and maintenance in the GE and GN groups were performed identically as described in the GA group. Intraoperative agents were titrated to achieve a BIS of 40–60.

### Data collection

Blood samples were taken from all patients before the operation (T0), 30 min after skin incision (T1), immediately after the operation (T2), 24 h (T3) and 72 h (T4) after an operation to test peripheral lymphocyte subtype analysis and evaluate the changes of immune function. At the same time points, the levels of plasma cortisol, epinephrine and norepinephrine, which reflect the stress level of patients^10^, were tested. The total dosage of opioids and propofol during the perioperative period, recovery time, mechanical ventilation time, extubation time and various adverse events in the three groups of patients were recorded. Visual Analogue Scale scores (VAS, range from 1 to 10; The larger the number, the stronger the pain experience) were recorded immediately after extubation and at 6 h, 12 h, 24 h, 48 h and 72 h after operation. The length of ICU stay, postoperative hospital stay and total hospitalization expenses were collected from the patients’ electronic medical records at the time of discharge. Patient satisfaction with perioperative pain management (range from 1 to 10; the larger the number, the more satisfied the patient is) was quantified by telephone 1 week after discharge.

The primary outcomes of the study were peripheral lymphocyte subtype analysis, including CD4, CD8, B lymphocyte and NK cell count. The secondary outcomes of the study were levels of plasma cortisol, epinephrine and norepinephrine.

### Calculation of sample size

To calculate the minimum sample size that would ensure the desired margin of error, we used the mean and standard deviation of the difference in NK cells of six patients selected from the electronic medical record system in 2021. At 24 h after the operation, the NK cells of groups GA and GE were 83.17 and 143.67, respectively; and the standard deviation was 55.25. Using PASS 11.0 software, we set an error of 0.01 and a power of 90%. Accordingly, 26 was the smallest acceptable patient sample size, and we increased the number of participants in each group to 30 to account for loss to follow-up.

### Statistical methods

Statistical software R (version 3.6.3; R core team, 2021) was used for statistical analysis. Quantitative data are expressed by means, standard deviation, median and interquartile range; and qualitative data are expressed by frequency and percentage. One way ANOVA, Chi square test and rank sum test (when necessary) were performed in non repetitive indicators. The comparison of repeated measurement indicators at different observation times among the three groups was performed using a linear mixed effect model (MEM) with a test level of *α* = 0.05. *P* < 0.05 was considered significant.

### Ethics approval

This clinical study was approved by the Ethics Committee of the General Hospital of Central Theater Command of People’s Liberation Army and passed the clinical trial registration application (ChiCTR2100047982).

### Consent to participate

Written informed consent was obtained from all subjects participating in the trial.

## Results

Ninety patients undergoing elective laparoscopic were selected. One patient in the GA group suffered from postoperative bleeding necessitating a second operation. One patient in the GE group had a postoperative infection. Therefore, they were excluded and a total of 88 patients performed the final analysis.

### Comparison of general clinical characteristics

There was no significant difference in gender, age, weight, operation type, operation time and amount of blood loss among the three groups, as shown in Table 1.

**Table 1:** Comparison of general clinical characteristics among the three groups.

	GA Group (*N* = 29)	GE Group (*N* = 29)	GN Group (*N* = 30)	*F*/*x*^2^	*P-*value
**Sex**				0.187	0.910
Male	16 (55.2)	16 (55.2)	18 (60.0)		
Female	13 (44.8)	13 (44.8)	12 (40.0)		
**Age (Y)**	59.55 ± 10.11	59.17 ± 7.98	58.93 ± 9.61	0.033	0.970
**Weight (kg)**	63.34 ± 9.12	63.55 ± 8.72	61.63 ± 9.10	0.407	0.667
**ASA classification**				0.158	0.924
II	22	21	23		
III	7	8	7		
**CCI**	10.50	11.81	11.94	0.792	0.673
**Tumor location**				0.631	0.960
Colon cancer	14 (48.3)	13 (44.8)	16 (53.3)		
Gastric cancer	9 (31.0)	9 (31.0)	9 (30.0)		
Rectal cancer	6 (20.7)	7 (24.1)	5 (16.7)		
**Operation time (min)**	267.07 ± 29.09	275.48 ± 36.62	259.77 ± 46.13	1.260	0.289
**Blood loss (ml)**	465.52 ± 76.89	479.31 ± 167.71	486.67 ± 147.94	0.181	0.834

*CCI* Charlson Comorbidity Index.

### Immune indexes

**Figure 1:**
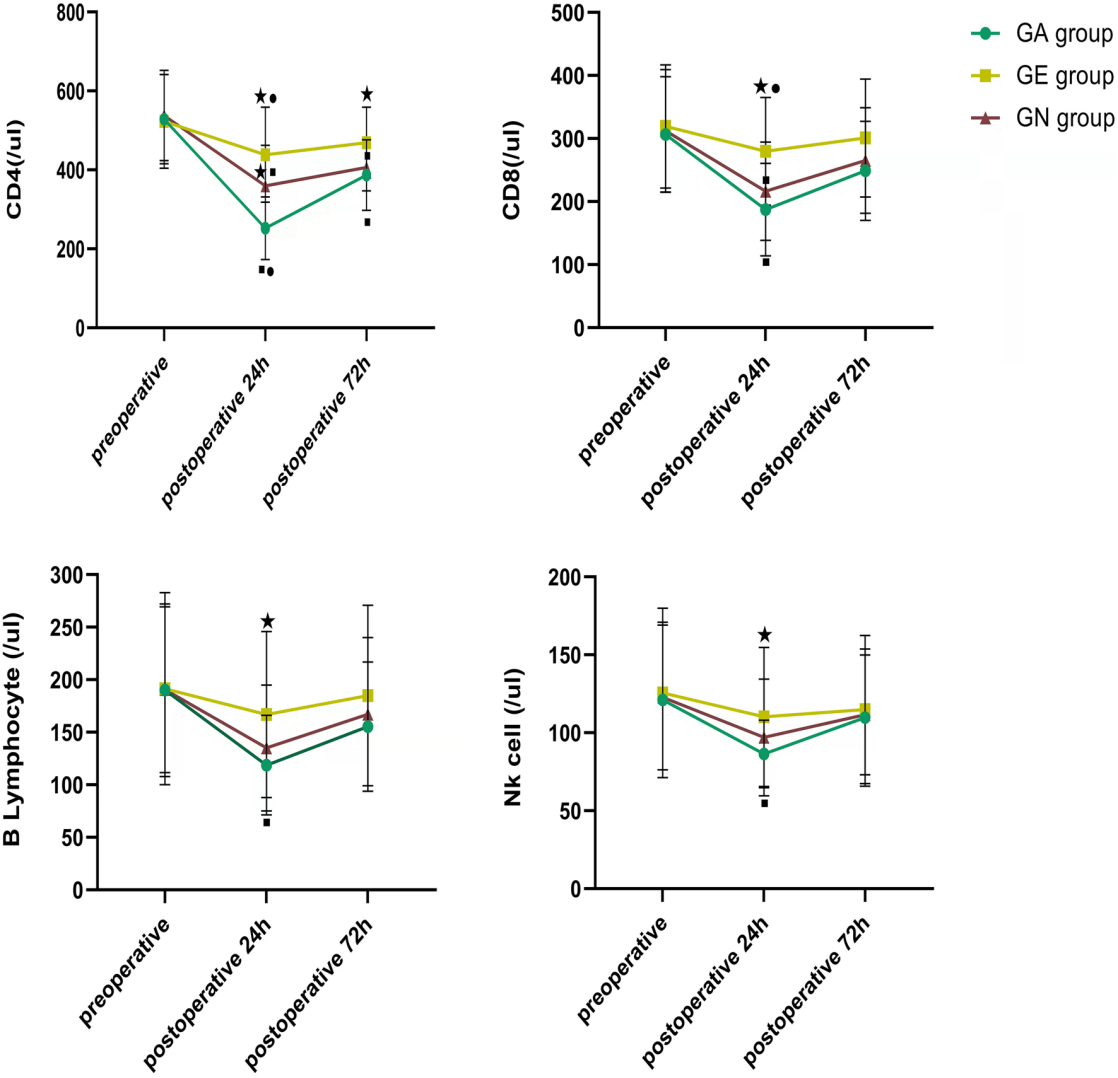
Comparison of immune indexes among the three groups. ★: Compared with the GA group, *P* < 0.05; ■: Compared with the GE group, *P* < 0.05; ●: Compared with the GN group, *P* < 0.05.

There was no difference in all immune indexes among the three groups at the T0 time point. At the T3 time point, the CD4 count in the EA group was higher than that in the NA and GA groups (*P* < 0.01), and that in the NA group was higher than that in GA group (*P* < 0.01). At the T4 time point, the CD4 count in the EA group was higher than that in the other two groups (*P* < 0.01), and there was no difference between the NA group and GA group (*P* > 0.05). At the T3 time point, the CD8 count in the EA group was higher than that in NA and GA groups (*P* < 0.01), and there was no difference between the NA group and GA group (*P* > 0.05). At the T4 time point, there was no difference among the three groups (*P* > 0.05). At the T3 time point, the B lymphocyte count in the EA group was higher than that in the GA group (*P* < 0.01), and there was no difference between the NA group and GA group (*P* > 0.05). At the T4 time point, there was no difference among the three groups (*P* > 0.05). At the T3 time point, the NK cell count in the EA group was higher than that in the GA group (*P* < 0.05), and there was no difference between the NA group and GA group (*P* > 0.05). At the T4 time point, there was no difference among the three groups (*P* > 0.05; Fig. 1).

### Comparison of stress index

**Figure 2:**
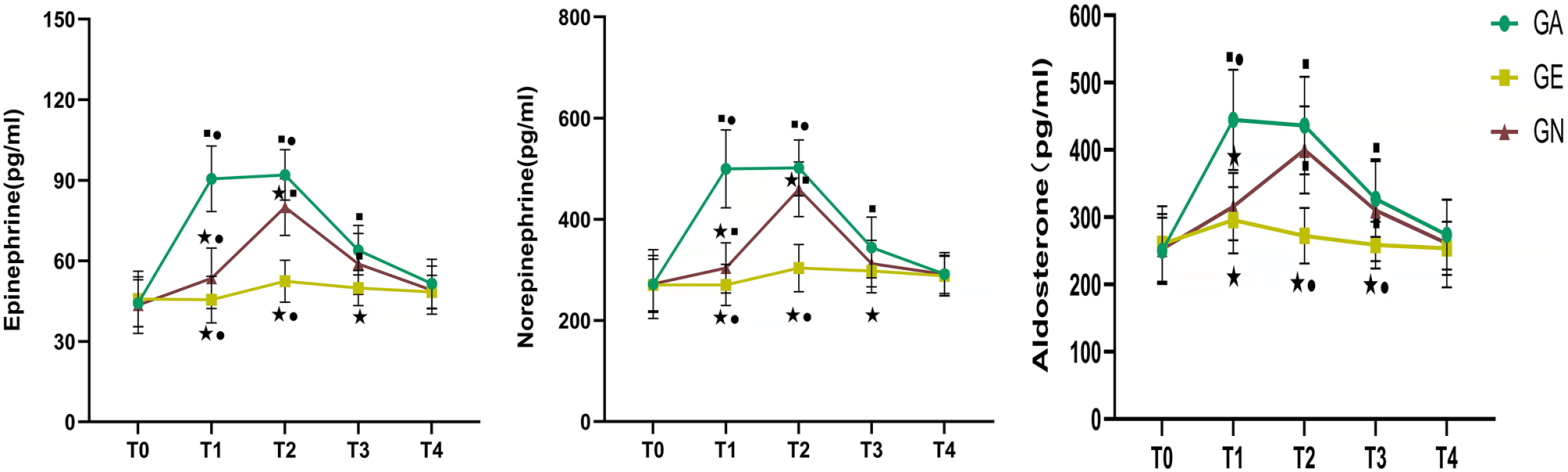
Comparison of stress indexes among the three groups. ★: Compared with the GA group, *P* < 0.05; ■: Compared with the GE group, *P* < 0.05; ●: Compared with the GN group, *P* < 0.05.

At T0 and T4, there was no difference in stress indices among the three groups (*P* > 0.05). Epinephrine levels at T1, T2, and T3 in the GE group were lower than those of the GN and GA groups, and lower than the GA group in the GN group (*P* < 0.01). Norepinephrine levels at T1 and T2 in the GE group were lower than those in GN and GA groups (P < 0.01, P < 0.01), and those in the GN group were lower than the GA group (*P* < 0.01). At the T3 time point, norepinephrine level in the GE group was lower than the GA group (*P* < 0.01), and there was no difference between GA and GN group (*P* > 0.05). Aldosterone levels in the GE and GN groups were lower than those in the GA group at the T1 time point (*P* < 0.01). At the T2 and T3 time points, aldosterone level in the GE group was lower than in the GN and GA groups (*P* < 0.01), and there was no difference between the GA and GN groups (*P* > 0.05; Fig. 2).

### Comparison of dosage of anesthetics

**Figure 3:**
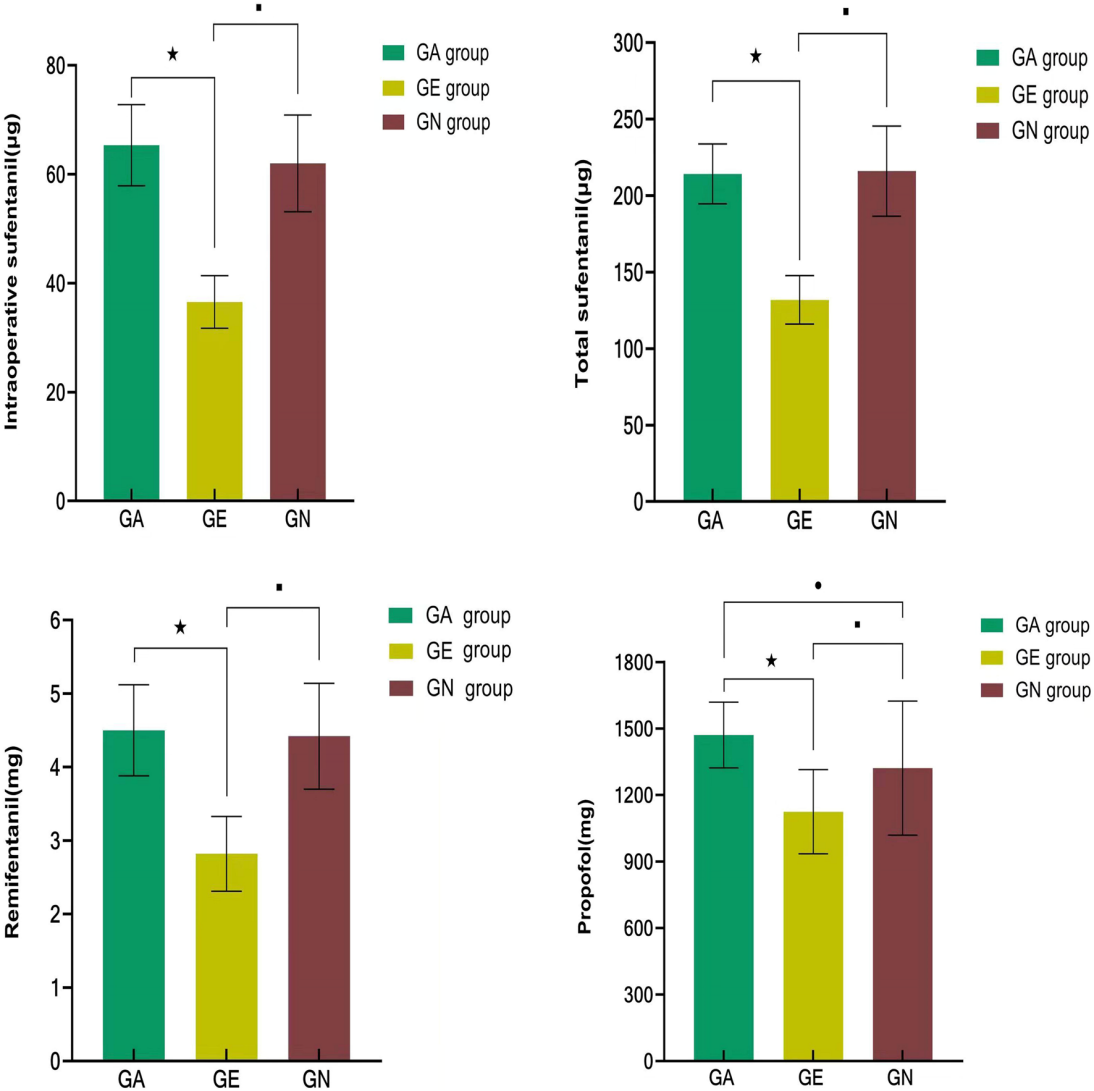
Comparison of the dosage of anesthetics among the three groups. ★: Compared with the GA group, *P* < 0.05; ■: Compared with the GE group, *P* < 0.05; ●: Compared with the GN group, *P* < 0.05.

Among the three groups, the intraoperative sufentanil dosage, total sufentanil dosage, remifentanil dosage and propofol dosage in the GE group were significantly lower than those in the GA group and GN group, and the above indices had no difference between the GA group and GN group (Fig. 3).

### Sensory level of epidural anesthesia and abdominal nerve block under ultrasound guidance

**Figure 4:**
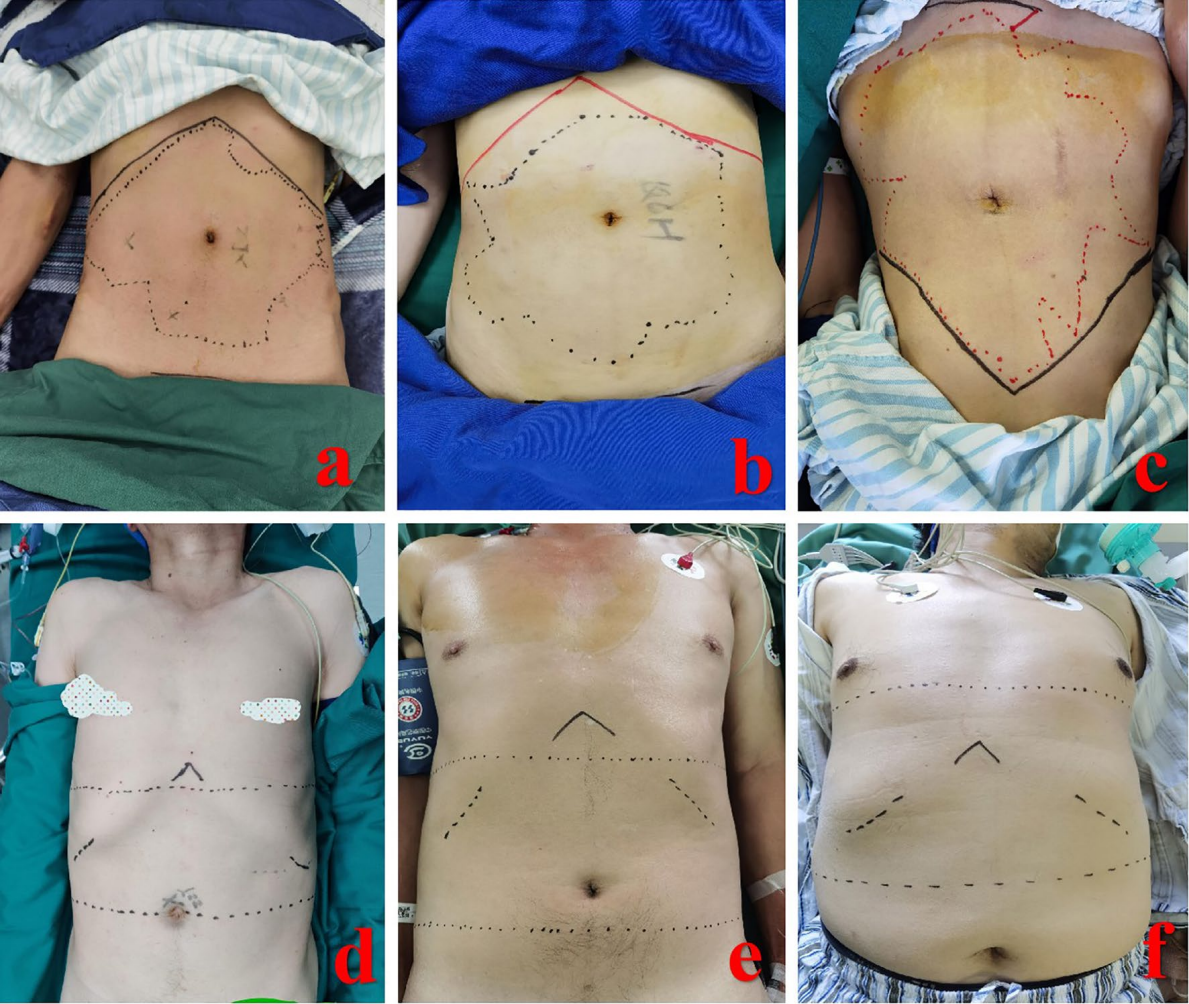
Sensory level of abdominal nerve block and epidural anesthesia with pinprick. (**a**–**c**) Sensory level of abdominal nerve block under ultrasound guidance; (**d**) epidural anesthesia at T11–T12; (**e**) epidural anesthesia at T12–L1; (**f**) epidural anesthesia at T9–T10.

The sensory level of abdominal nerve block under ultrasound guidance (Fig. 4a–c) and epidural anesthesia at different puncture points (Fig. 4d at T11–T12, Fig. 4e at T12–L1, Fig. 4f at T9–T10) is shown in Fig. 4.

### Comparison of analgesic effect

**Figure 5:**
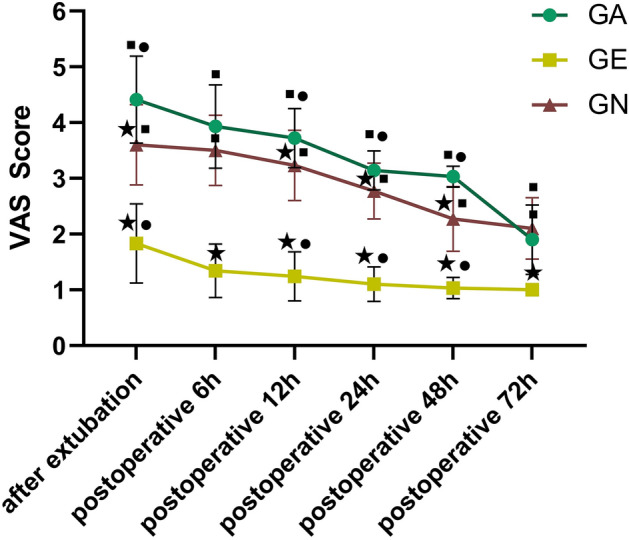
Comparison of postoperative VAS scores among the three groups. ★: Compared with the GA group, *P* < 0.05; ■: Compared with the GE group, *P* < 0.05; ●: Compared with the GN group, *P* < 0.05.

The VAS scores immediately after extubation and at 6 h, 12 h, 24 h, 48 h and 72 h after operation in the GE group were improved significantly more than those in the GA group and GN group (*P* < 0.01). At 12 h, 24 h and 48 h after the operation, the VAS score of the GN group was better than that of the GA group (*P* < 0.05), and there was no difference at other times (*P* > 0.05; Fig. 5). The effective pressing times and total pressing times of the postoperative analgesic pump in the GE group were significantly less than those in GA and GN groups (*P* < 0.01), but there was no difference between the GA and GN groups (*P* > 0.05). In terms of patient satisfaction with pain management, patient satisfaction (10 point Likert scale) in the GE group was significantly better than that in GA and GN groups, and that in the GN group was better than that in the GA group (Table 2).

**Table 2:** Comparison of the press times of the analgesic pump and pain management satisfaction.

	GA Group (*N* = 29)	GE Group (*N* = 29)	GN Group (*N* = 30)	*F*/*x*^2^	*P*-value
Effective press times of analgesic pump^†^	21.00 (17.00, 23.00)	0.00 (0.00, 2.00)^a^	14.00 (12.25, 15.00)^b^	69.977	**< 0.001**
Total press times of analgesic pump^†^	49.00 (35.00, 53.00)	0.00 (0.00, 3.00)^a^	25.00 (19.25, 31.25)^b^	70.124	**< 0.001**
Pain management satisfaction (1–10)	3.10 ± 0.82	8.21 ± 0.94^a^	5.07 ± 1.01^a,b^	222.893	**< 0.001**

^a^Compared with the GA group, *P* < 0.05; ^b^Compared with the GE group, *P* < 0.05.

^†^The index did not conform to the normal distribution. The index is expressed as the median and interquartile range, and the rank sum test was used for comparison between groups.

Effective press times of analgesic pump: in order to ensure the safety of patients, there is a locking time after pressing the analgesic pump to avoid excessive drug entering the body. Pressing beyond the locking time is defined as effective pressing.

Significant values are in bold.

### Comparison of perioperative adverse events and time efficiency

The incidence of intraoperative hypertension in the GE group was lower than that in the GA group (*P* < 0.05), and there was no difference between the GE group and GN group. There was no difference in the incidence of intraoperative hypotension, perioperative shivering and perioperative hypoxemia among the three groups. The extubation time, ICU stay and duration of hospitalization in the GE group were shorter than in the GA group and GN group (*P* < 0.05). The length of ICU stay in the GN group was shorter than that in the GA group (*P* < 0.05). The total hospitalization expenses in the GE group were lower than those in the GA group (*P* < 0.05; Table 3).

**Table 3:** Comparison of perioperative adverse events and time efficiency among the three groups.

	GA Group (*N* = 29)	GE Group (*N* = 29)	GN Group (*N* = 30)	*F*/*x*^2^	*P*-value
Intraoperative hypertension	10 (34.5)	1 (3.4)^a^	8 (26.7)	8.942	**0.011**
Intraoperative hypotension	5 (17.2)	7 (24.1)	4 (13.3)	1.182	0.554
Perioperative shivering	3 (10.3)	3 (10.3)	3 (10.0)	0.002	1.000
Perioperative hypoxemia	1 (3.4)	1 (3.4)	0 (0.0)	–	0.542
Extubation time (min)	50.97 ± 14.28	16.69 ± 5.91^a^	45.70 ± 9.26^b^	91.774	**< 0.001**
ICU stay (hour)	27.79 ± 6.36	16.45 ± 4.76^a^	22.60 ± 4.33^a,b^	34.431	**< 0.001**
Duration of hospitalization (day)	18.28 ± 2.31	15.24 ± 1.84^a^	17.37 ± 2.58^b^	13.662	**< 0.001**
Total hospitalization expenses (10,000 yuan)	9.66 ± 0.90	8.92 ± 0.58^a^	9.38 ± 0.96	5.721	**0.005**

^a^Compared with the GA group, P < 0.05; ^b^Compared with the GE group, P < 0.05.

Intraoperative hypertension was defined as the patient's blood pressure increased by more than 30% of the basic blood pressure, or the systolic blood pressure was ≥ 140 mmHg and/or the diastolic blood pressure was ≥ 90 mmHg; Intraoperative hypotension was defined as a systolic pressure less than 90 mmHg or reduction greater than 20% of baseline; Perioperative shivering was defined as involuntary body shivering and perioperative hypoxemia was defined as blood oxygen saturation less than 90%.

Significant values are in bold.

## Discussion

In the present study, the principle finding was that both GA combined with epidural anesthesia and GA combined with nerve block has protective effects on postoperative immune function compared with GA alone, while GA combined with epidural anesthesia performs better. Our results are consistent with those reported elsewhere^7–9^.

### Perioperative immune dysfunction

Immune function is an important means for the body to resist external diseases, among which T lymphocyte subsets are the most important and are the core element for maintaining normal immune function. CD4^+^ cells are involved in the expression of surface molecules and the secretion of cytokines. CD8^+^ cells are cytotoxic T lymphocytes. Their changes indicate changes in immune system function, where a significant decline usually means serious diseases and unfavorable prognosis^11^. NK cells are the first line of immunity defense for tumor cells; they can nonspecifically identify and eliminate tumor cells; and inhibit tumor formation, growth, spread and metastasis^12^, thereby reducing the formation of metastasis during the survival of small tumors or tumor cells^13^.

During the perioperative period, various factors, including hypothermia, blood transfusion and painful stimulation, can inhibit immune function, resulting in immunosuppression. In particular, anesthetics^3,5^ and perioperative stress response have significant immunosuppressive effects. A sizable body of literature has suggested that opioids, inhaled anesthetics, stress response and other factors can significantly inhibit immune function^3,5^. Our study also showed that the number of CD4, CD8, total B lymphocytes, and NK cells decreased significantly after gastrointestinal surgery under general anesthesia, suggesting that the patient may be predisposed to an immunosuppressive state. During the operation, due to surgical methods such as incision, manipulation, and electric cauterization, it is possible to freely disseminate tumor cells. If the immune function decreases at this time, it is posited that an increased possibility of postoperative recurrence and/or distant metastasis can occur.

### Effect of stress response on immune function

Surgical stress occurs throughout the whole perioperative period, and has been proven to significantly inhibit the function of the cell-mediated immune system, especially the inhibition of NK and T cell activities^14^. We believe that the degree of immunosuppression is directly proportional to the degree of surgical stress.

General anesthesia can reduce the degree of surgical trauma by inhibiting the activity of the central nervous system^4^, but it has no significant inhibitory effect on the nociceptive signal transmission along the somatic and sympathetic nerves. Additionally, the compromise in immune function, activation of the stress response, and hemodynamic fluctuations caused by surgical trauma are not ideal^15^. However, epidural block can suppress the spinal cord nerve impulse generated by surgical stimulation, reduce its effect on the hypothalamus–pituitary–adrenal cortex axis, inhibit sympathetic nerve activity, and alleviate the cellular immunosuppression caused by surgical stress^16,17^.

Our results also showed that the there was less intraoperative hypertension in the GE group than in the GA group, and the levels of plasma epinephrine, norepinephrine and cortisol were significantly lower than those in the GA and GN groups, confirming that general anesthesia combined with epidural anesthesia could better reduce the stress level of patients undergoing gastrointestinal tumor resection.

Postoperative pain management of the GE group was better than that of the GA and GN groups, which also attenuated the stress response. Better postoperative pain management could be a mechanism by which GA combined with epidural anesthesia exerts better immune function protection.

### Effect of anesthetics on immune function

Sedative and analgesic drugs often used in general anesthesia have dual effects on immune function. Anesthetics can inhibit the perioperative stress response, including inhibiting the HPA axis and reducing the level of catecholamine release, thereby exerting an immunoprotective effect^18,19^. However, almost all anesthetic drugs modulate the function of the immune system, either directly or indirectly^3,20^. Opioids also affect the adaptive immune system and the underlying molecular mechanisms including leukocyte apoptosis and inhibition of leukocyte cell production^21^. Opioids mediate an immunosuppressive response facilitating tumor dissemination^22^. Studies associate opioids with poorer cancer outcomes in pancreatic^23^ and colorectal^24^ cancer as well as prostate cancer^25^ in humans. However, propofol has been demonstrated to have immune protective effects^26^.

In our study, general anesthesia combined with epidural anesthesia was shown to significantly reduce intraoperative/total sufentanil dosage and intraoperative remifentanil dosage, which could be the mechanism by which this anesthetic technique exerts immunoprotection.

Others have suggested that GA combined with peripheral nerve blocks can significantly protect postoperative immune function^8^. Our data did not demonstrate this same effect in our subset. We think that even experienced anesthesiologists operating under the guidance of ultrasound cannot guarantee the sufficient block range of peripheral nerve block, as shown in Fig. 3. Also, only a small part of the perioperative pain of laparoscopic gastrointestinal tumor resection comes from the incision pain, while more pain comes from visceral pain and inflammatory pain. Epidural anesthesia can effectively reduce this pain, but peripheral nerve block cannot achieve a similar effect, which may be due to the lack of a sympathetic block.

## Limitations

Our study has several limitations. First, our sample size is relatively small. Two patients with postoperative bleeding and infection were excluded from the study, which may have a certain impact on the test results. Second, our observation time for all patients’ immune function was up to 3 days after the operation. The long-term effects of different anesthetic methods on postoperative immune function need to be further studied. Third, patients undergoing different surgeries have been recruited. The stress response, as well as postoperative pain may be different.

## Conclusions

Compared with GA combined with nerve block or GA alone, GA combined with epidural anesthesia has better protective effects on postoperative immune function. Its possible mechanisms include reducing perioperative stress response and reducing perioperative opioid dosage.

## Data Availability

All data generated or analyzed during this study are included in this article.

## References

[1] Momosaki K, Ishibashi N, Yoshida S (2014). Effect of preoperative administration of methylprednisolone and ulinastatin on tumor cell metastasis after surgical stress. Kurume Med. J..

[2] Zhu S, Zhao Y, Quan Y, Ma X (2021). Targeting myeloid-derived suppressor cells derived from surgical stress: The key to prevent post-surgical metastasis. Front. Surg..

[3] Schneemilch CE, Schilling T, Bank U (2004). Effects of general anaesthesia on inflammation. Best Pract. Res. Clin. Anaesthesiol..

[4] Cruz FF, Rocco P, Pelosi P (2021). Immunomodulators in anesthesia. Curr. Opin. Anaesthesiol..

[5] Snyder GL, Greenberg S (2010). Effect of anaesthetic technique and other perioperative factors on cancer recurrence. Br. J. Anaesth..

[6] Zhou M, Liu W, Peng J, Wang Y (2021). Impact of propofol epidural anesthesia on immune function and inflammatory factors in patients undergoing gastric cancer surgery. Am. J. Transl. Res..

[7] Xu ZZ, Li HJ, Li MH (2021). Epidural anesthesia-analgesia and recurrence-free survival after lung cancer surgery: A randomized trial. Anesthesiology.

[8] Cong X, Huang Z, Zhang L (2021). Effect of different anaesthesia methods on perioperative cellular immune function and long-term outcome in patients undergoing radical resection of esophageal cancer: A prospective cohort study. Am. J. Transl. Res..

[9] Zhang W, Cong X, Zhang L (2020). Effects of thoracic nerve block on perioperative lung injury, immune function, and recovery after thoracic surgery. Clin. Transl. Med..

[10] Meng Z, Gao C, Li X (2021). Effects of combined epidural anesthesia and general anesthesia on cognitive function and stress responses of elderly patients undergoing liver cancer surgery. J. Oncol..

[11] Bolla G, Tuzzato G (2003). Immunologic postoperative competence after laparoscopy versus laparotomy. Surg. Endosc..

[12] Vitale M, Cantoni C, Pietra G, Mingari MC, Moretta L (2014). Effect of tumor cells and tumor microenvironment on NK-cell function. Eur. J. Immunol..

[13] Lugli E, Marcenaro E, Mavilio D (2014). NK cell subset redistribution during the course of viral infections. Front. Immunol..

[14] Elenkov IJ, Chrousos GP (2002). Stress hormones, proinflammatory and antiinflammatory cytokines, and autoimmunity. Ann. N. Y. Acad. Sci..

[15] Liu W, Wu L, Zhang M, Zhao L (2019). Effects of general anesthesia with combined epidural anesthesia on inflammatory response in patients with early-stage gastric cancer undergoing tumor resection. Exp. Ther. Med..

[16] Song P, Dong T, Zhang J, Li J, Lu W (2017). Effects of different methods of anesthesia and analgesia on immune function and serum tumor marker levels in critically ill patients. Exp. Ther. Med..

[17] Zhu R, Xiang J, Tan M (2020). Effects of different anesthesia and analgesia on cellular immunity and cognitive function of patients after surgery for esophageal cancer. Minerva Chir..

[18] Kurosawa S, Kato M (2008). Anesthetics, immune cells, and immune responses. J. Anesth..

[19] Cruz FF, Rocco PR, Pelosi P (2017). Anti-inflammatory properties of anesthetic agents. Crit. Care.

[20] Welden B, Gates G, Mallari R, Garrett N (2009). Effects of anesthetics and analgesics on natural killer cell activity. AANA J..

[21] Ninković J, Roy S (2013). Role of the mu-opioid receptor in opioid modulation of immune function. Amino Acids.

[22] Lennon FE, Moss J, Singleton PA (2012). The μ-opioid receptor in cancer progression: Is there a direct effect. Anesthesiology.

[23] Chen W, Xu Y, Zhang Y, Lou W, Han X (2021). Positive impact of intraoperative epidural ropivacaine infusion on oncologic outcomes in pancreatic cancer patients undergoing pancreatectomy: A retrospective cohort study. J. Cancer.

[24] Zimmitti G, Soliz J, Aloia TA (2016). Positive impact of epidural analgesia on oncologic outcomes in patients undergoing resection of colorectal liver metastases. Ann. Surg. Oncol..

[25] Lec PM, Lenis AT, Golla V (2020). The role of opioids and their receptors in urological malignancy: A review. J. Urol..

[26] Irwin MG, Chung C, Ip KY, Wiles MD (2020). Influence of propofol-based total intravenous anaesthesia on peri-operative outcome measures: A narrative review. Anaesthesia.

